# Unraveling the Complexity: A Case of Systemic Lupus Erythematosus, Warm Autoimmune Hemolytic Anemia, and Autoimmune Cholangitis

**DOI:** 10.7759/cureus.95692

**Published:** 2025-10-29

**Authors:** Rupam Sharma, Kevin T Dao, Jose Garcia-Corella, Harmanjeet Dhillon, Ishaan Kalha, Stanley Kim, William R Stull, Greti Petersen

**Affiliations:** 1 Internal Medicine, David Geffen School of Medicine, Los Angeles, USA; 2 Internal Medicine, University of California at Los Angeles (UCLA) Kern Medical, Bakersfield, USA; 3 Gastroenterology, University of California at Los Angeles (UCLA) Kern Medical, Bakersfield, USA; 4 Hematology and Oncology, University of California at Los Angeles (UCLA) Kern Medical, Bakersfield, USA; 5 Pathology, University of California at Los Angeles (UCLA) Kern Medical, Bakersfield, USA

**Keywords:** autoimmune cholangitis, autoimmune hepatitis, multidisciplinary approach, overlap syndrome (os), systemic lupus erythematosus, warm autoimmune hemolytic anemia

## Abstract

Systemic lupus erythematosus (SLE), warm autoimmune hemolytic anemia (WAIHA), and autoimmune cholangitis (AIC) are three distinct autoimmune conditions, each posing its own clinical challenges. The coexistence of these diseases in a single patient, however, is exceedingly rare and presents a complex diagnostic and therapeutic dilemma. SLE is an autoimmune disease that can cause a constellation of pathologies that affect various organs. WAIHA involves the premature destruction of red blood cells due to immunoglobulin (IgG) autoantibodies, leading to hemolytic anemia. AIC is characterized by features of both autoimmune hepatitis and cholestatic liver disease, which adds further complexity to this overlap syndrome. Herein presented is a case of a patient with simultaneous SLE, WAIHA, and AIC, highlighting the overlapping symptoms, diagnostic challenges, and the multifaceted approach to management. The case underscores the importance of a comprehensive, multidisciplinary approach to address the interplay of multiple autoimmune disorders, given the risk of therapeutic conflicts and disease progression.

## Introduction

In many instances, having one autoimmune disease can be a unique case depending on the disease in question. However, having three different types of autoimmune diseases that are unrelated is quite an anomaly. Systemic lupus erythematosus (SLE), warm autoimmune hemolytic anemia (WAIHA), and autoimmune cholangitis (AIC) are distinct autoimmune conditions, each with a unique pathophysiology and clinical presentation. However, the coexistence of these diseases within a single patient presents a rare and complex medical dilemma. SLE is a systemic autoimmune disease characterized by a variety of differing symptoms that can make the diagnosis fairly difficult. In the majority of cases, both immunological testing and clinical assessment are necessary in determining the diagnosis [1]. WAIHA, on the other hand, involves the premature destruction of red blood cells due to autoantibodies, predominantly of the immunoglobulin G (IgG) type, that react optimally at body temperature [2]. A variety of precipitating factors can induce the precipitation of WAIHA. However, what complicates matters is that patients with SLE can also have anemia. Although the main key is that patients with WAIHA would have abnormal hemolysis blood work and elevated indirect bilirubin. This can be further complicated in patients with autoimmune hepatitis or AIC. In fact, overlap syndromes themselves are generally conditions where two or more autoimmune conditions occur in conjunction, such as SLE and rheumatoid arthritis or scleroderma and myositis, etc. AIC in and of itself is referred to as an “overlap syndrome,” which is a rare condition where features of autoimmune hepatitis and cholestatic liver disease coexist, further complicating the clinical picture [3].

In short, the intersection of these autoimmune conditions presents a diagnostic and therapeutic challenge due to the overlapping symptomatology, immunological interactions, and treatment complications. This case report explores a patient who presented with concurrent SLE, WAIHA, and AIC, discussing the intricate interplay of these conditions and the complexities involved in managing their simultaneous progression.

This article was previously presented as a poster presentation at AFMR Western Region 2024 and as a poster presentation at UCLA Solomon Scholar 2024

## Case presentation

This patient is a 41-year-old woman with a recent diagnosis of cirrhosis with unknown etiology who presented to the emergency department (ED) with a chief complaint of subjective fevers and rigors for one week and a productive cough with brownish sputum for three days. She also stated having symptoms of severe fatigue, muscle wasting, unquantified unintentional weight loss, and yellowish discoloration of her skin over the last year prior to her presentation. She otherwise denied any other symptoms at this current time. Of note, one month prior, the patient was evaluated at an outside facility for abdominal pain and was diagnosed with cirrhosis. She additionally underwent esophagogastroduodenoscopy (EGD) at that time. The findings revealed trace esophageal varices in the distal esophagus and mild patchy gastric erythema and edema. The biopsy was negative for *Helicobacter pylori*.

**Figure 1:**
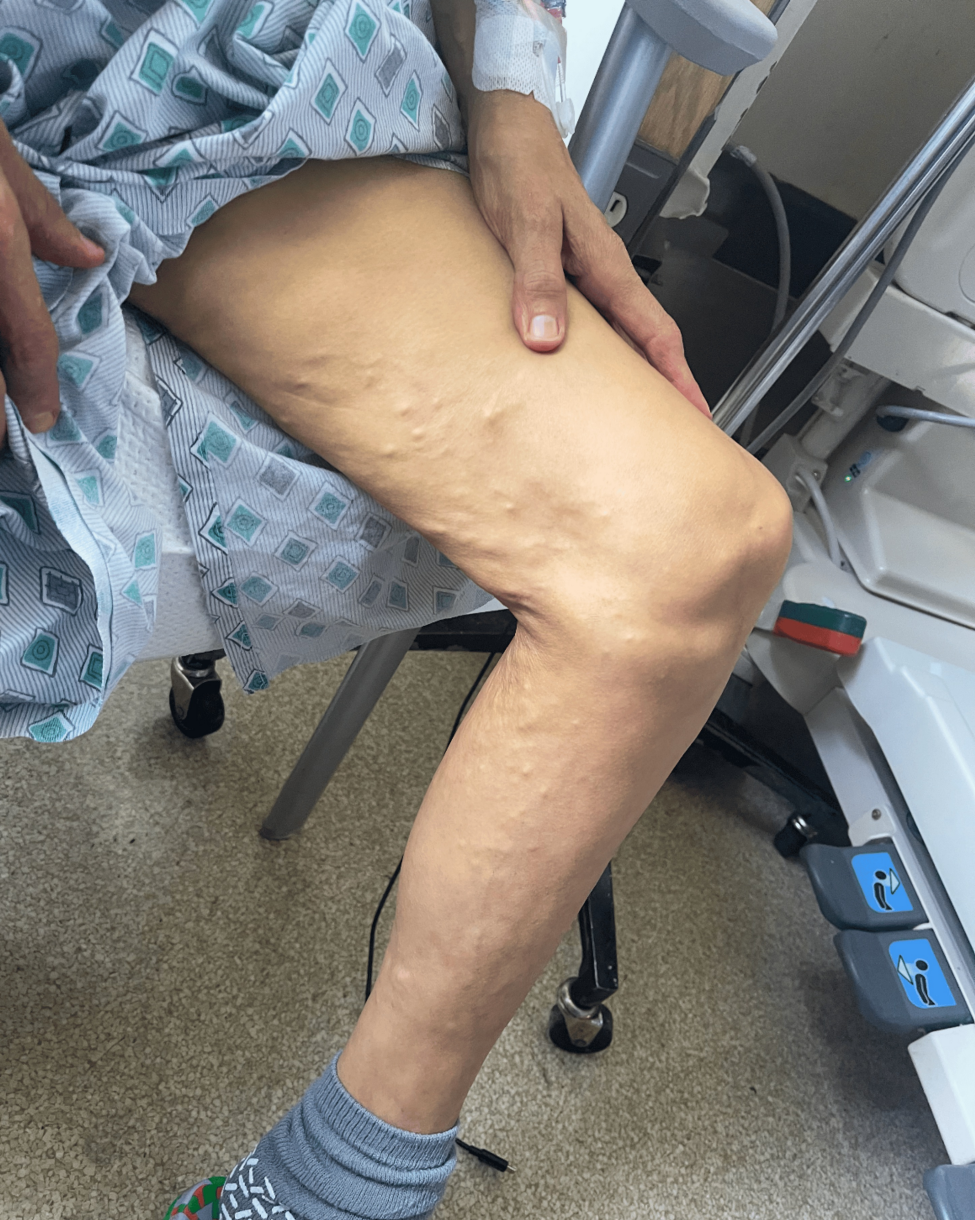
Physical Exam Findings

Upon arrival at the emergency department, the patient was febrile at 39.4 degrees Fahrenheit, tachypneic with a respiratory rate of 24 breaths per minute and hypotensive with a blood pressure of 89/47 mmHg. The physical exam showed a cachectic-appearing woman with diffuse jaundice and scleral icterus; the abdomen was distended and positive for fluid wave. Additionally, small, approximately 1-2 mm diffuse skin-colored hard deposits were noted on the bilateral lower extremities, more in the inner thighs; no other cutaneous findings were noted (Figure 1).

**Figure 2:**
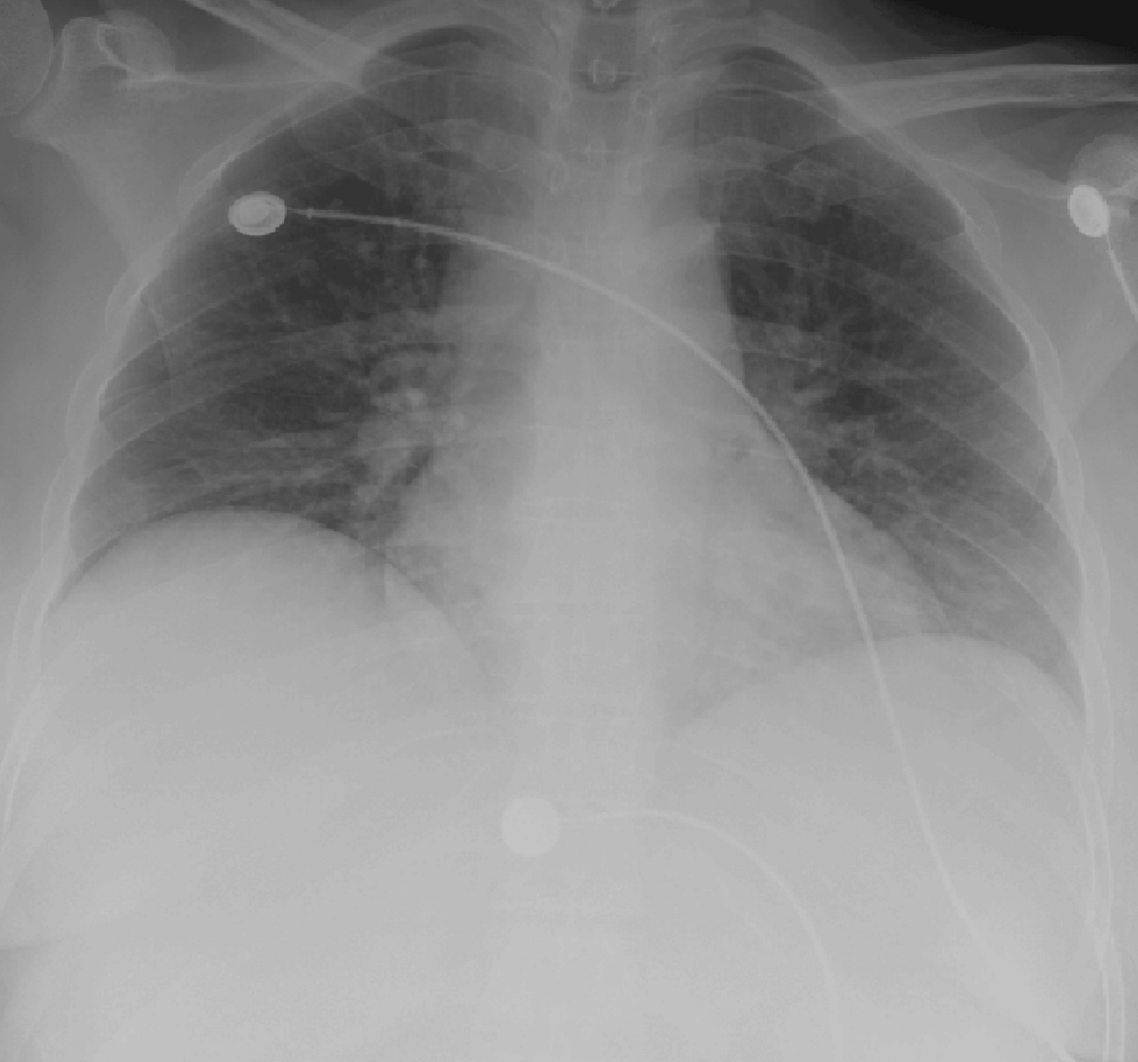
Chest X-Ray Suboptimal inspiration, anteroposterior (AP) magnification, accentuate cardiac silhouette. Mild peribronchial cuffing. Slight basilar atelectasis, worse on right. Compared to prior, no definite acute infiltrates, large pleural effusions, pulmonary edema, pneumothorax noted. Minimal degenerative changes.

**Figure 3:**
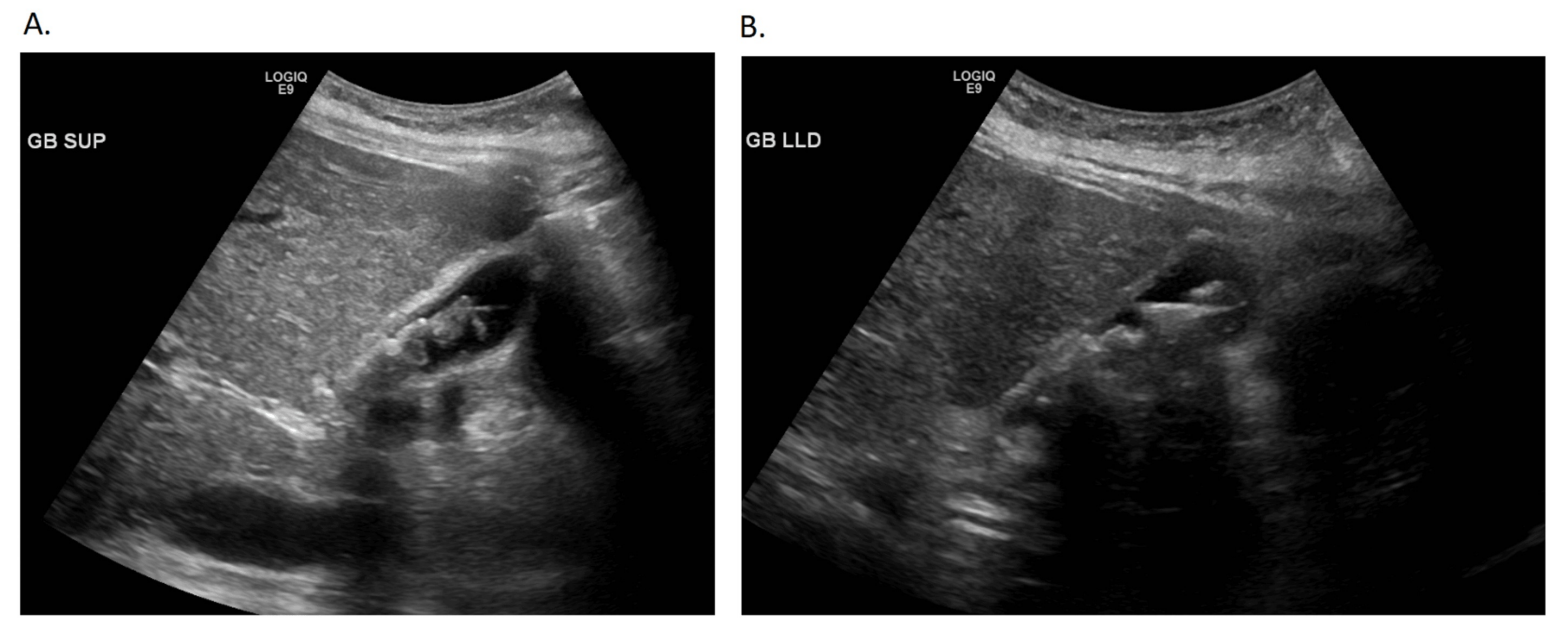
Ultrasound of Abdomen Gallbladder showed echogenic lobulated mobile contents that may represent tumefactive sludge and/or cholelithiasis. Mild gallbladder wall thickening. Common bile duct in porta region about 4 mm does not appear dilated, could not be traced distally and no positive Murphy's sign noted. No pericholecystic fluid.

**Figure 4:**
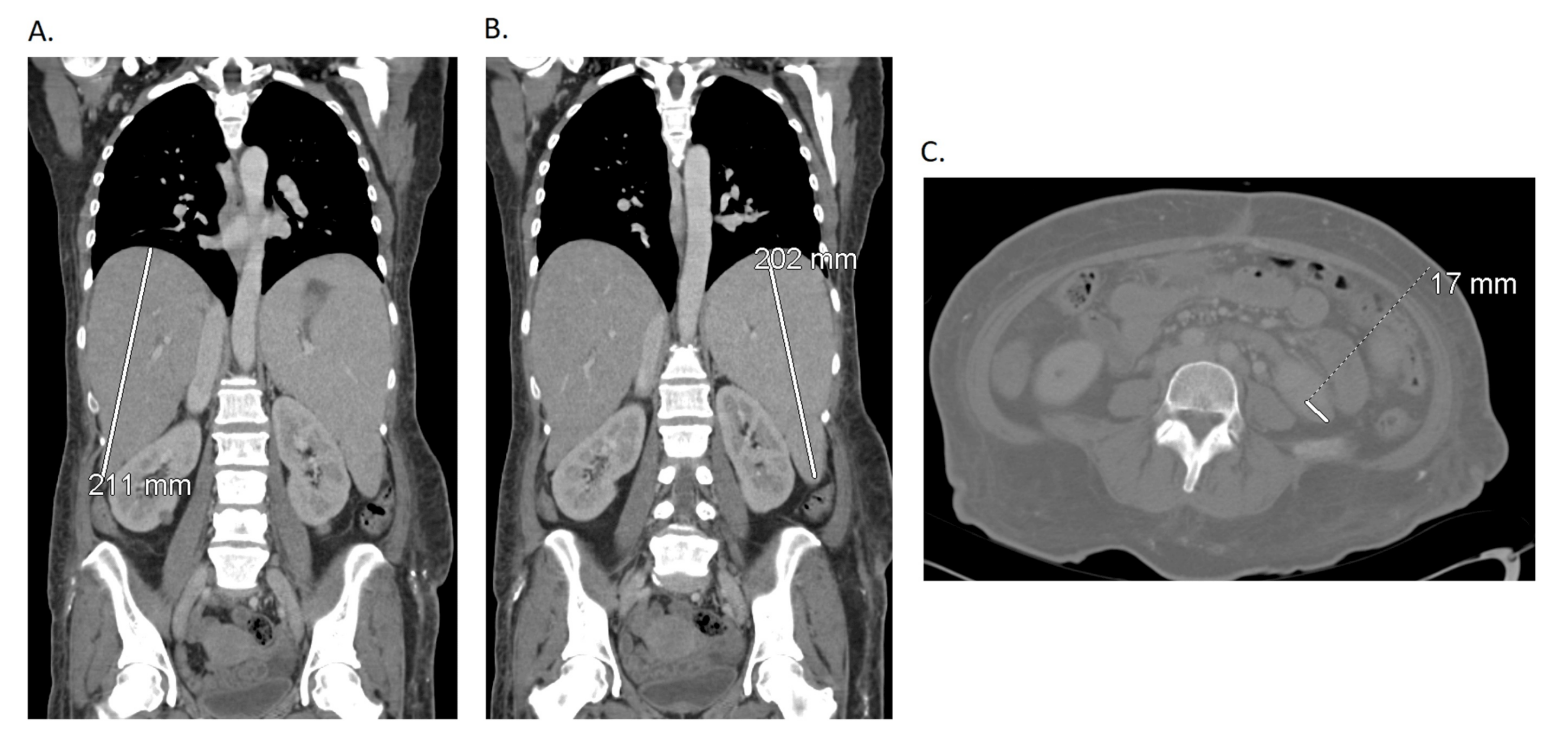
Computed tomography (CT) of Abdomen and Pelvis with Contrast (A) Enlarged liver with suspected underlying hepatocellular steatosis versus hepatocellular disease with the liver measuring up to 21 cm in length. No focal lesion is definitively seen. (B) Moderate to severe splenomegaly measuring 21 cm in length. No focal splenic lesion. (C) Hyperdense cystic lesion originating from the lower pole the left kidney measuring up to 17 mm in size.

Initial blood work was done (Table 1) and a respiratory Biofire viral panel (bioMérieux, Marcy-l'Étoile, France) was also performed, which resulted in a positive human metapneumovirus finding but was otherwise unremarkable. The hepatitis panel was noted to be unremarkable, and further imaging showed an unremarkable chest x-ray (Figure 2). Abdominal ultrasound showed fatty and/or hepatocellular changes with a mildly enlarged liver with echogenic lobulated mobile constant in the gallbladder (Figure 3). This was further confirmed by computed tomography (CT) of the abdomen and pelvis with contrast (Figure 4A-C). The patient was initiated on treatment for sepsis with fluid resuscitation and broad-spectrum antibiotics, including vancomycin and piperacillin/tazobactam. During hospitalization, an extensive laboratory workup was ordered (Table 2), and outside records were obtained to further evaluate the etiology of the patient’s cirrhosis.

**Table 1:** Initial Blood Work

Parameter	Value	Reference
Sodium	128 mmol/L	136-145 mmol/L
Potassium	3.8 mmol/L	3.5-5.1 mmol/L
Chloride	108 mmol/L	98-107 mmol/L
Calcium (corrected)	9.6 mg/dL	8.5-10.1 mg/dL
Magnesium	1.7 mg/dL	1.8-2.4 mg/dL
Phosphorus	2.6 mg/dL	2.5-4.9 mg/dL
Alanine transaminase	29 units/L	13-61 units/L
Aspartate transaminase	91 units/L	15-37 units/L
Alkaline phosphatase	281 units/L	45-117 units/L
Direct bilirubin	8.4 mg/dL	0-0.2 mg/dL
Total bilirubin	10.9 mg/dL	0-1 mg/dL
Lactate dehydrogenase	548 unit/L	84-246 unit/L
Albumin	1.7 g/dL	3.4-5 g/dL
Erythrocyte sedimentation rate	>100 mm/h	<20 mm/h
Lactic acid	3.5 mmol/L	0.4-2 mmol/L
White blood count (WBC)	5.5×10^3/mcL	4.5-11×10^3/mcL
Hemoglobin (Hgb)	6.2 g/dL	13.2-17.4 g/dL
Mean corpuscular value (MCV)	106.8 HI	80-98 HI
Platelet	152×10^3/mcL	150-450 x10^3/mcL
Neutrophils	78.2%	50–75%
Lymphocytes	15.3%	20-45%
Bands	0%	<12%
Monocytes	0.4 %	2-12 %
Eosinophils	0%	<6%
Absolute neutrophil	9.1×10^3/mcL	1.8-7.7×10^3/mcL
Absolute lymphocyte	0.9×10^3/mcL	1.2–4.5×10^3/mcL
Absolute monocyte	0.4×10^3/mcL	0.1-1×10^3/mcL
Absolute eosinophil	0×10^3/mcL	<0.7×10^3/mcL

**Table 2:** Further Blood Work P-ANCA: Perinuclear anti-neutrophil cytoplasmic antibody; DAT: direct antiglobulin test.

Parameter	Value	Reference
Antinuclear antibody (ANA)	Positive	Negative
ANA titer	1:320	<1:40
P-ANCA	1:640	<1:20
DNA (ds) Antibody	20 international unit/mL	< 4 international unit/mL
Anti-mitochondrial	Negative	Negative
Smooth muscle antibody	Negative	Negative
Anti-C antibody	Positive	Negative
Gamma-glutamyl transferase (GGT)	168 unit/L	3-55 unit/L
Alpha fetoprotein tumor marker	2.8 ng/mL	<8 ng/mL
Ceruloplasmin	36 mg/dL	18-53 mg/dL
DAT - Poly-specific	Positive	Negative
DAT - IgG	Positive	Negative
DAT - C3d	Positive	Negative
Complement C3c	38 mg/dL	83-193 mg/dL
Complement C4c	10 mg/dL	15-57 mg/dL
Haptoglobin	<8 mg/dL	43-212 mg/dL
Vitamin B12	1155 pg/mL	193-986 pg/mL
Iron level	119 mcg/dL	50-170 mcg/dL
Total iron binding capacity (TIBC)	156 mcg/dL	250-450 mcg/dL
Ferritin	145 ng/mL	8-252 ng/mL
Immunoglobulin A (IgA)	262 mg/dL	47-310 mg/dL
Immunoglobulin G (IgG)	5319 mg/dL	600-1640 mg/dL
Immunoglobulin M (IgM)	729 mg/dL	50-300 mg/dL

**Figure 5:**
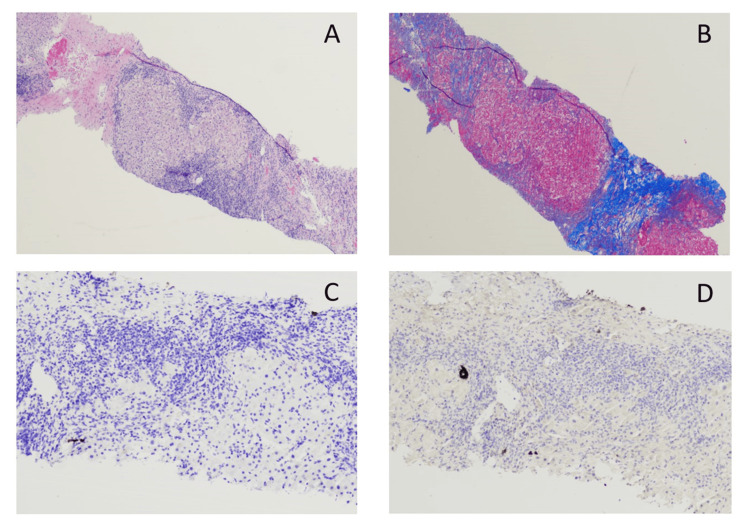
Liver Biopsy Pathology (A) H&E staining showing fibrotic bands surrounding liver nodule. (B) Trichrome staining revealing significant fibrosis consistent with advanced cirrhosis (blue). (C,D) Ductopenia as evident by decreased and scattered small-duct uptake of cytokeratin 7 and 19, respectively.

Liver biopsy confirmed fibrosis (Figure 5A,B) and revealed cholestasis and significant ductopenia with decreased and scattered cytokeratin 7 and 19 uptake in the small biliary ducts, specifically (Figure 5C,D). The patient’s fibrosis score was 0.95 (high) with a necrosis inflammatory activity score of 0.41 (high), grade A1 to A2, F4 cirrhosis, and subsequently the patient was initiated on treatment for Coombs-positive WAHA with intravenous (IV) immunoglobulin (Ig) 1 g/kg/day for two days with a plan for continuing prednisone and a consideration for rituximab IV infusion upon discharge; however, the patient fortunately didn’t require rituximab. The patient also had serum protein electrophoresis (SPEP) findings showing an M spike with an abnormal protein band of 3.8 g/dL. Subsequently, she underwent immunofixation, which revealed two separate M spike's IgG kappa 132.9 mg/dL and IgG lambda 120.7 mg/dL after which the patient underwent CT-guided bone marrow biopsy to rule out underlying plasma cell dyscrasia such as multiple myeloma. The bone marrow biopsy did not reveal any monoclonal plasma cells and only revealed increased erythrocyte series consistent with hemolytic anemia. A repeat immunoglobulin panel throughout hospitalization showed high IgG at 6295 mg/dL secondary to IVIg treatment and underlying lupus. Throughout hospitalization, the patient was started on prednisone 60 mg daily with a plan to continue for three weeks and trimethoprim/sulfamethoxazole for pneumocystis jiroveci pneumonia (PJP) prophylaxis due to long-term steroids. On hospital day 18, the patient was discharged home in a stable condition with a plan to follow-up with outpatient hematology, rheumatology, gastroenterology, and internal medicine.

Since discharge from the hospital, the patient has been followed up in the outpatient hematology/oncology clinic, with significant improvement noted in the patient’s overall clinical status. She is currently on prednisone 18 mg a day, which is being tapered very slowly by 1 mg/week. Her lab values have improved, with bilirubin level at 3.2 mg/dL and hemoglobin level increasing to 10.6 g/dL. She is currently also being evaluated for liver transplantation at a higher level of care for hepatobiliary surgery. Overall, her liver function is improving now with careful steroid therapy.

## Discussion

This patient presented with a myriad of symptoms, acutely with sepsis secondary to pneumonia, initially thought to be unrelated to her past medical history of cirrhosis of unknown etiology. However, after further investigation, a complex autoimmune process with multiple organ involvement was revealed.

SLE is a multi-organ autoimmune disease primarily diagnosed through both clinical findings and immunological blood work and can be triggered through a variety of different environmental factors [4]. Based on the patient’s clinical presentation as well as blood work, a variety of different autoimmune etiologies were identified, such as SLE, AIHA, microscopic polyangiitis, autoimmune hepatitis (AIH), and primary biliary cholangitis (PBC). The patient’s positive DNA (ds) antibody as well as low complement levels (Table 2) made the suspicion of SLE prominent. What is interesting is that the patient had also had an elevated perinuclear anti-neutrophil cytoplasmic antibody (P-ANCA), which did raise the concern for microscopic polyangiitis; however, on further review, there have been studies associated with P-ANCA and SLE [5]. The anemia as well as elevated indirect bilirubin and lactate dehydrogenase raise a concern for hemolysis. However, what can complicate matters is that SLE can be associated with hematological manifestations such as anemia [6].

AIHA can sometimes be linked to other autoimmune disorders, making it crucial to recognize it within a broader diagnostic spectrum and consider various differential diagnoses [7]. A study revealed that 77% of secondary warm AIHA cases occur in women, with an average onset at 36 years, reflecting the higher prevalence of autoimmune diseases in women within this age group [8]. This suggests a notable connection between warm AIHA and autoimmune conditions, particularly in Hispanic populations, as observed in our patient. In this case, our patient developed WAIHA along with SLE. She fortunately responded well to the treatment with IVIg and steroids. The patient continued to follow up outpatient with hematology with significant improvement in her hemoglobin levels and overall weakness.

What also stood out was not only the presence of cirrhosis of unknown cause in a patient with no past medical history, but also the advanced stage of the disease, with abdominal ultrasound and CT scan revealing a nodular cirrhotic liver with evidence of portal hypertension, including splenomegaly and collateral vasculature. A non-invasive fibrosis test with elastography confirmed this diagnosis, showing advanced cirrhosis and high inflammatory activity [9].

Considering the negative hepatitis viral panel, as well as the lack of risk factors suggesting metabolic or alcohol-associated liver disease, the next step in the approach to evaluate the patient’s cirrhosis etiology was to obtain and analyze the pattern of autoimmune antibodies present. This autoantibody panel was not specific enough to suggest previously suspected vasculitis, autoimmune hepatitis (AIH) or primary biliary cholangitis (PBC); however, the pattern of elevated antinuclear antibody (ANA) and double-stranded DNA (dsDNA), along with the clinical presentation, provided enough evidence to diagnose SLE. Despite this diagnosis, there is a low correlation between SLE and cirrhosis [10], raising suspicion for an alternative etiology within the same autoimmune spectrum. The presence of multiple autoimmune conditions in a single patient is well understood due to the shared pathogenesis of these diseases [11]. This combination of factors prompted the obtention of a liver biopsy to review the architecture and accurately diagnose the etiology.

The liver biopsy and results showed that the findings are consistent with small duct cholangiopathy, which is a feature that is found in primary biliary cirrhosis (PBC) and small duct primary sclerosing cholangitis (sdPSC) and is consistent with the suspected autoimmune etiology that matched this patient’s clinical presentation; however, it could not be explained by a single condition. AIC has been described as an immune-mediated destruction of the bile ducts that includes a spectrum of findings seen in sdPSC, antimitochondrial antibody (AMA) negative PBC, and IgG4-mediated sclerosing cholangitis and can be considered a variant of AIH [12]. These clinical, immunological, and microscopic findings can overlap, creating what is known as an AIC-variant syndrome [13].

## Conclusions

In conclusion, the coexistence of SLE, WAIHA, and autoimmune cholangitis (AIC) presents a rare and challenging clinical scenario. This case illustrates the complex interplay between multiple autoimmune diseases, where overlapping symptoms and immunopathology create diagnostic difficulties and complicate treatment decisions. Clinicians themselves should be aware of such overlap syndromes to ensure effective management, which requires a multidisciplinary approach in order to balance the risks of immunosuppressive therapy with the progression of each disease. As autoimmune overlap syndromes are relatively uncommon, further research and case studies are essential to improve our understanding of their pathogenesis and guide optimal treatment strategies.
